# Yttrium-90 radioembolization for unresectable hepatocellular carcinoma: predictive modeling strategies to anticipate tumor response and improve patient selection

**DOI:** 10.1007/s00330-022-08585-x

**Published:** 2022-03-01

**Authors:** Willie Magnus Lüdemann, Johannes Kahn, Daniel Pustelnik, Juliane Hardt, Georg Böning, Martin Jonczyk, Holger Amthauer, Bernhard Gebauer, Bernd Hamm, Gero Wieners

**Affiliations:** 1 Department of Radiology, Campus Virchow Klinikum, Charité, Augustenburger Platz 1, 13353 Berlin, Germany; 2 Department of Biometry, Epidemiology and Information Processing, WHO Collaborating Centre for Research and Training for Health in the Human-Animal-Environment Interface, University of Veterinary Medicine Hannover (Foundation), Buenteweg 2, 30559 Hanover, Germany; 3 Department of Nuclear Medicine, Augustenburger Platz 1, 13353 Berlin, Germany

**Keywords:** Carcinoma, hepatocellular, Treatment outcome, Survival analysis, Tumor burden, Yttrium radioisotopes

## Abstract

**Objectives:**

This study aims to better characterize potential responders of Y-90-radioembolization at baseline through analysis of clinical variables and contrast enhanced (CE) MRI tumor volumetry in order to adjust therapeutic regimens early on and to improve treatment outcomes.

**Methods:**

Fifty-eight HCC patients who underwent Y-90-radioembolization at our center between 10/2008 and 02/2017 were retrospectively included. Pre- and post-treatment target lesion volumes were measured as total tumor volume (TTV) and enhancing tumor volume (ETV). Survival analysis was performed with Cox regression models to evaluate 65% ETV reduction as surrogate endpoint for treatment efficacy. Univariable and multivariable logistic regression analyses were used to evaluate the combination of baseline clinical variables and tumor volumetry as predictors of ≥ 65% ETV reduction.

**Results:**

Mean patients’ age was 66 (SD 8.7) years, and 12 were female (21%). Sixty-seven percent of patients suffered from liver cirrhosis. Median survival was 11 months. A threshold of ≥ 65% in ETV reduction allowed for a significant (*p* = 0.04) separation of the survival curves with a median survival of 11 months in non-responders and 17 months in responders. Administered activity per tumor volume did predict neither survival nor ETV reduction. A baseline ETV/TTV ratio greater than 50% was the most important predictor of arterial devascularization (odds ratio 6.3) in a statistically significant (*p* = 0.001) multivariable logistic regression model. The effect size was strong with a Cohen’s *f* of 0.89.

**Conclusion:**

We present a novel approach to identify promising candidates for Y-90 radioembolization at pre-treatment baseline MRI using tumor volumetry and clinical baseline variables.

**Key Points:**

*• A decrease of 65% enhancing tumor volume (ETV) on follow-up imaging 2–3 months after Y-90 radioembolization of HCC enables the early prediction of significantly improved median overall survival (11 months vs. 17 months, p = 0.04).*

*• Said decrease in vascularization is predictable at baseline: an ETV greater than 50% is the most important variable in a multivariable logistic regression model that predicts responders at a high level of significance (p = 0.001) with an area under the curve of 87%.*

**Supplementary Information:**

The online version contains supplementary material available at 10.1007/s00330-022-08585-x.

## Introduction

Yttrium-90 (Y-90) radioembolization is a minimally invasive intra-arterial therapy for unresectable liver tumors such as advanced hepatocellular carcinoma (HCC) or liver metastases [1]. Selected patients with intermediate to advanced stage, unresectable HCC benefit substantially more from Y-90 radioembolization than others which demands further investigation to improve patient selection [2].

HCC is the third most common cause of tumor-related death worldwide [3]. Despite the widespread use of screening programs, 60–70% of HCCs are detected at intermediate and advanced stages when curative treatment approaches such as surgical therapy or ablation are precluded [4, 5]. Y-90 radioembolization is performed interchangeably with TACE for intermediate stage HCC at some centers or as a second-line therapy following TACE failure for intermediate and advanced stage HCC [1, 6, 7]. During radioembolization, radionuclides such as Yttrium-90 or Holmium-166 embedded in microspheres are applied via branches of the hepatic artery. Given the predominantly arterial blood supply of HCC nodules, the microspheres accumulate in the tumor micro-vasculature and emit high-energy, low-penetration beta radiation to the tumor [8]. The technique proved non-inferior to TACE in a series of phase 2 trials [6, 9–12]. Both the SIRveNIB and the SARAH trials, the first randomized controlled phase 3 trials confirming the safety and efficacy of Y-90 radioembolization in patients with locally advanced HCC, showed Y-90 radioembolization to be better tolerated than chemotherapy with sorafenib but failed to prove a superiority of Y-90 radioembolization in terms of overall and progression-free survival [13, 14]. Recent evidence underlines that treatment response is strongly dependent on the actual, deployed radiation dose within the tumor which, in turn, is highly correlated with the arterial tumor vascularization before treatment [15–18].

This study aims to better characterize potential responders of Y-90 radioembolization through analysis of contrast enhanced (CE) MRI tumor volumetry. Regression analysis is used to control for confounding clinical variables and for building an exemplary multivariable prediction model of MR morphologic treatment response at baseline.

## Materials and methods

### Study cohort

**Fig. 1:**
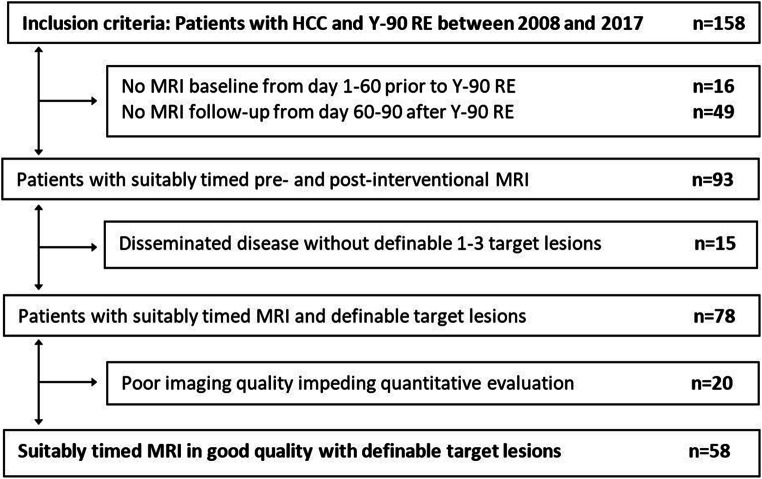
Flowchart of the patient selection process

This retrospective single-institution study was approved by the institutional ethics committee. Written informed consent was waived due to retrospective character of the study. All therapies were endorsed by an interdisciplinary tumor board in accordance with current recommendations [19, 20]. All patients who underwent their first Y-90 radioembolization session between 10/2008 and 02/2017 were evaluated for inclusion. Exclusion criteria were missing baseline MRI in a 60-day timeframe before radioembolization or missing follow-up MRI between day 60 and 90 afterwards, disseminated disease that impeded segmentation, and poor imaging quality. The patient selection process is presented as a flowchart in Fig. 1.

### Y-90 radioembolization protocol

Board-certified radiologists (B.G., G.W., F.S., D.S., all with > 10 years of experience, and D.G., with 1 year of experience after certification) performed the interventions according to a standard protocol (Supplement 1). At our institution, Y-90 radioembolization is performed with Yttrium 90 (Y-90) resin microspheres (SIR-Spheres®, Sirtex Medical Pty. Ltd.). The prescribed activity of Y-90 resin microsphere was determined according to body surface area (BSA) method. There was no dose reduction performed for patients with signs of liver cirrhosis.

### Imaging technique

Every patient received cross-sectional imaging before and after Y-90 radioembolization. According to the standard protocols at our institution, CE MRI was performed with a 1.5-T (Siemens Avanto or Aera) imaging unit. A phased-array torso coil and 0.1 mL per kilogram body weight of intravenous dinatrium gadoxetat (Eovist/Primovist, Bayer Healthcare) were used. The MRI protocol included breath-hold unenhanced and CE T1-weighted 3D fat-suppressed spoiled gradient-echo imaging (section thickness, 2.5 mm; receiver bandwidth, 64 kHz; flip angle, 10°) in the arterial phase (delay of 15 s after bolus tracking), the portal venous phase (delay of 70 s), the delayed phase (delay 3 min), and the hepatobiliary excretion phase (delay of 20 min after administration). Arterial phase was used for volumetric evaluation.

### Quantitative measurement of arterial tumor vascularization on CE MRI

**Fig. 2:**
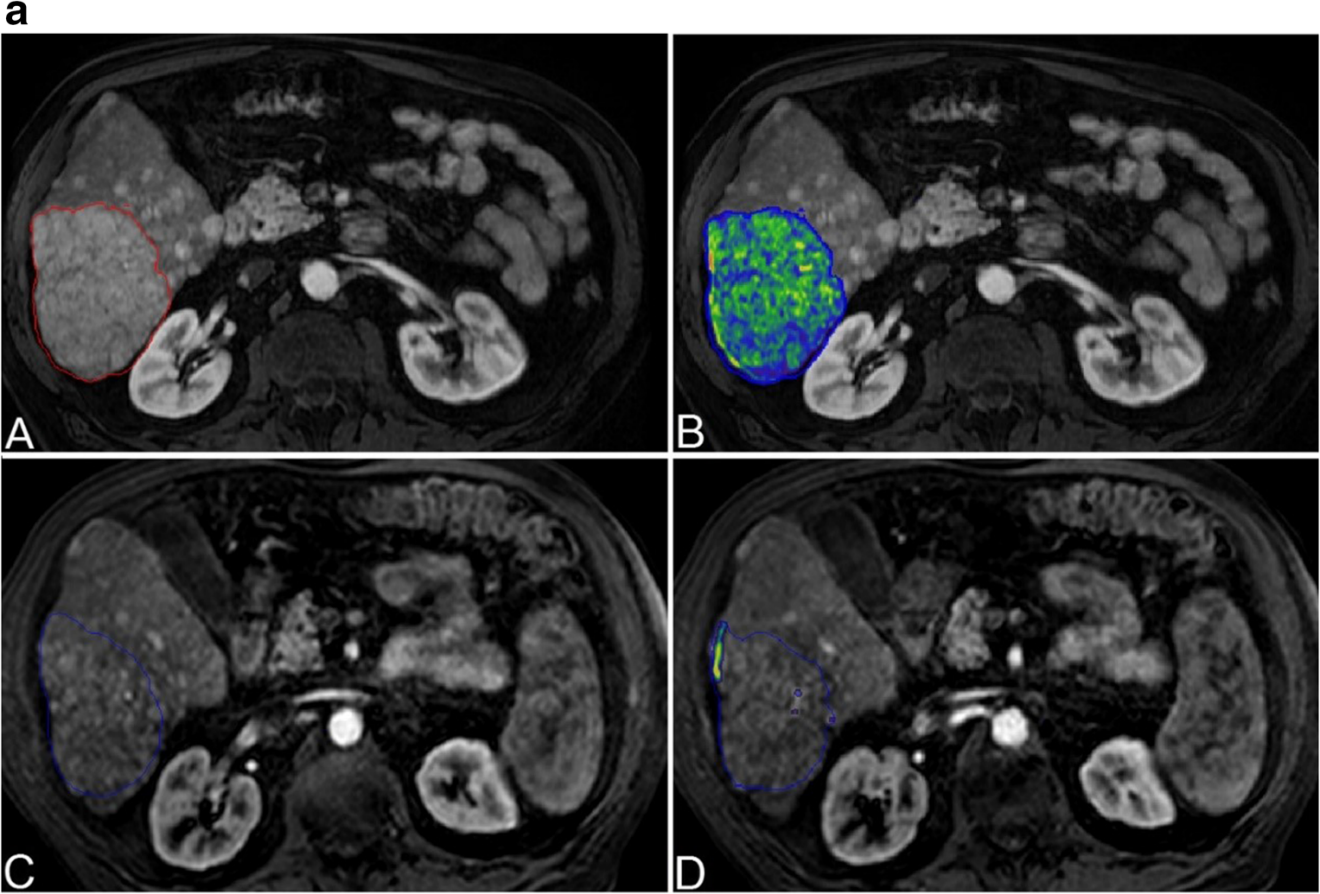

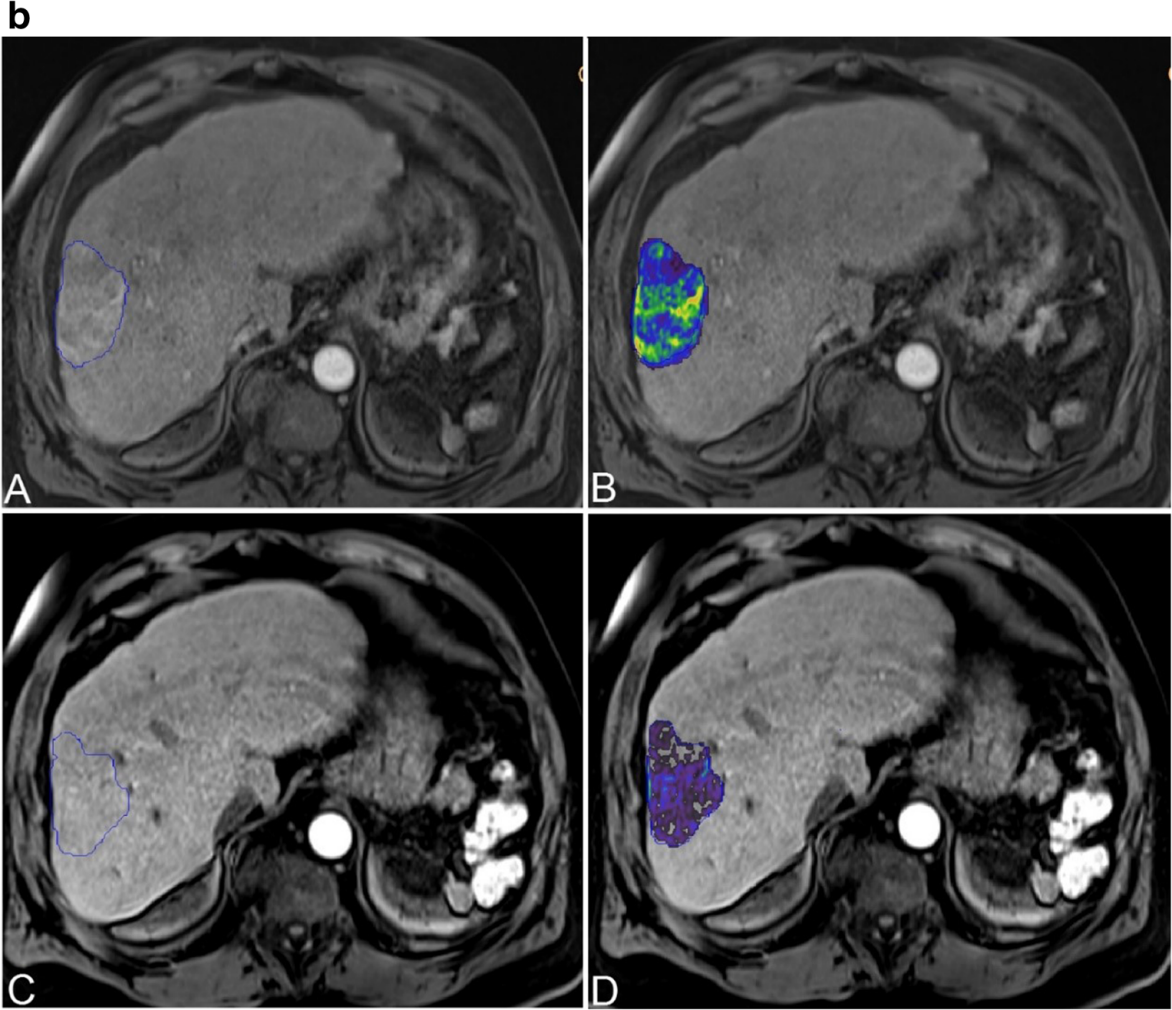
Quantification of total tumor volume and enhancing tumor volume on baseline and follow-up imaging. **a** Patient with highly vascularized HCC. Images only show representative tumor measurements in one layer. Target lesions were volumetrically assessed. A Preinterventional MRI: tumor tissue appears highly vascularized compared to surrounding liver parenchyma. B Colored enhancing tumor volume/total tumor volume (ETV/TTV) overlay. C Postinterventional MRI: the tumor appears to be de-vascularized after treatment. D ETV/TTV overlay. **b** Patient with intermediately vascularized HCC. A Preinterventional MRI: Intermediate vascularization of tumor; relative arterial vascularization is color-coded in (B). C Postinterventional MRI: a tumor with initially intermediate vascularization loses hyper-vascularization compared to surrounding liver tissue. D ETV/TTV overlay

For all measurements, a semi-automated quantification software (Philips IntelliSpace Portal) was used (Fig. 2). Accuracy and reproducibility were previously demonstrated [7, 8, 13, 17–19]. Two readers with 6 and 7 years of experience supervised the measurements. A signal intensity greater two standard deviations than the average signal intensity measured within a region of interest (ROI) of 10 × 10 × 10 mm in the musculus erector spinae was defined an arterial vascularization. As most patients had multifocal disease with more than 3 lesions in 97% of the cases, a maximum of three dominant lesions were analyzed and combined as surrogate total tumor volume (TTV) and enhancing tumor volume (ETV). In each case, the same lesions were measured before and after treatment. The measurements are given in milliliters. Relative vascularization was calculated as the ratio of ETV and TTV and expressed as percentage.

### Variables for the prediction of survival and MR morphologic response

To assess MR morphologic treatment response, we employed the quantitative European Association for the Study of the Liver (qEASL) criteria of ≥ 65% decrease in enhancing tumor volumes on follow-up MRI [21]. Alongside qEASL response, we evaluated measurements and derived indices of TTV and ETV at baseline as well as routinely available clinical variables as potential predictors of patient survival. Clinical variables were demographic data of gender and age, underlying liver disease, previous systemic or local treatments, and the radioembolization approach with respect to whole liver vs. sequential therapy or dose reduction. Baseline imaging and clinical variables were also assessed as predictors of qEASL response.

### Statistical analysis

All statistical analyses were conducted with IBM SPSS STATISTICS, version 25 (IBM Corporation). Testing for normality was performed with the Shapiro–Wilk test, and skewed data were transformed with the natural logarithm for further analysis. Normally distributed, continuous data are presented with mean and standard deviation and compared with the two-sample *t* test. Non-normally distributed data are expressed as median and interquartile range (IQR) and compared with the Mann–Whitney *U* test or the Wilcoxon signed-rank test. Agreement of two was measured with the Pearson correlation coefficient (PCC) statistic. Receiver operating characteristic (ROC) curves and Youden’s *J* statistic were used to evaluate thresholds for binary classifiers. Survival is reported as median with a 95% confidence interval and was analyzed with the Cox proportional hazard model, Kaplan-Meier curves, and the log-rank test. Median overall survival (mOS) was defined from the date of the first Y-90 radioembolization session until death. Patients who were either lost to follow-up or alive at the end-of-observation date were censored. To identify predictors for MRI morphologic treatment response at baseline, the effects of the abovementioned variables were investigated with logistic regression analysis. Model building for logistic regression analysis was informed by the resulting impact on Nagelkerke’s pseudo-*R*^2^. Multivariable Cox regression models were compared with the log-likelihood ratio statistic. Regression coefficients, hazard ratios (HR), and odds ratios (OR) as well as *p* values are presented. As this is an explorative study, no adjustment for multiple testing was applied. Thus, *p* values < 0.05 are reported as significant, but are only given as an orientation and to be interpreted cautiously and not as confirmatory.

## Results

### Patient characteristics

Fifty-eight patients were included for tumor volumetry and further analysis in predictive modeling. Patient inclusion process is shown in Fig. 1. Patient characteristics are summarized in Table 1 and contrasted with the 100 excluded patients in Supplement 2. About 65% of the patients had Barcelona clinic liver cancer (BCLC) stage B; liver cirrhosis was present in 67% and portal vein thrombosis in 26% of the included patients. All patients had an Eastern Cooperative Oncology Group (ECOG) performance status of 0–1.

**Table 1:** Patient characteristics at baseline

Patient characteristics		
Demographics		
Number of patients	58	
Age (years), mean (SD)	66 (8.7)	
Sex: male/female (% male)	46/12 (79%)	
Median survival in months (95% CI)*	11 [8–14]	
Liver disease		
BCLC stage	B	38 (66%)
	C	20 (34%)
Child Pugh Score	A	56 (97%)
	B	2 (3%)
	C	0
Cirrhosis	39/58 (67%)	
Hepatitis (B, C)	21/58 [6, 15] (36%)	
Portal vein thrombosis	Proximal occlusion	7 (12%)
	Proximal partial occlusion	1 (2%)
	Left	3 (5%)
	Right	4 (7%)
Previous treatments (possible multiple therapies)		
Resection	10 (17%)	
TACE	16 (28%)	
Brachytherapy	15 (26%)	
RFA	4 (7%)	
PEI	1 (2%)	
Sorafenib	16 (28%)	
Other systemic therapies	2 (3%)	
Specifics of Y-90 radioembolization		
Sequential/whole liver therapy	35/23	
Hepatopulmonary shunt %, mean (SD)	8.6% (4.7%)	
Median tumor volumes in mL (IQR)	209 (326)	
Median activity in mBq (IQR)	1.7 (0.53)	

*Kaplan–Meier estimator

*BCLC*, Barcelona Clinic liver cancer stage (BCLC); *CI*, confidence interval; *ECOG*, Eastern Cooperative Oncology Group performance (ECOG); *IQR*, interquartile range; *PEI*, percutaneous ethanol injection; *RFA*, radiofrequency ablation; *SD*, standard deviation; *TACE*, transarterial chemoembolization

### MRI tumor segmentation at baseline and 3 months after treatment

**Fig. 3:**
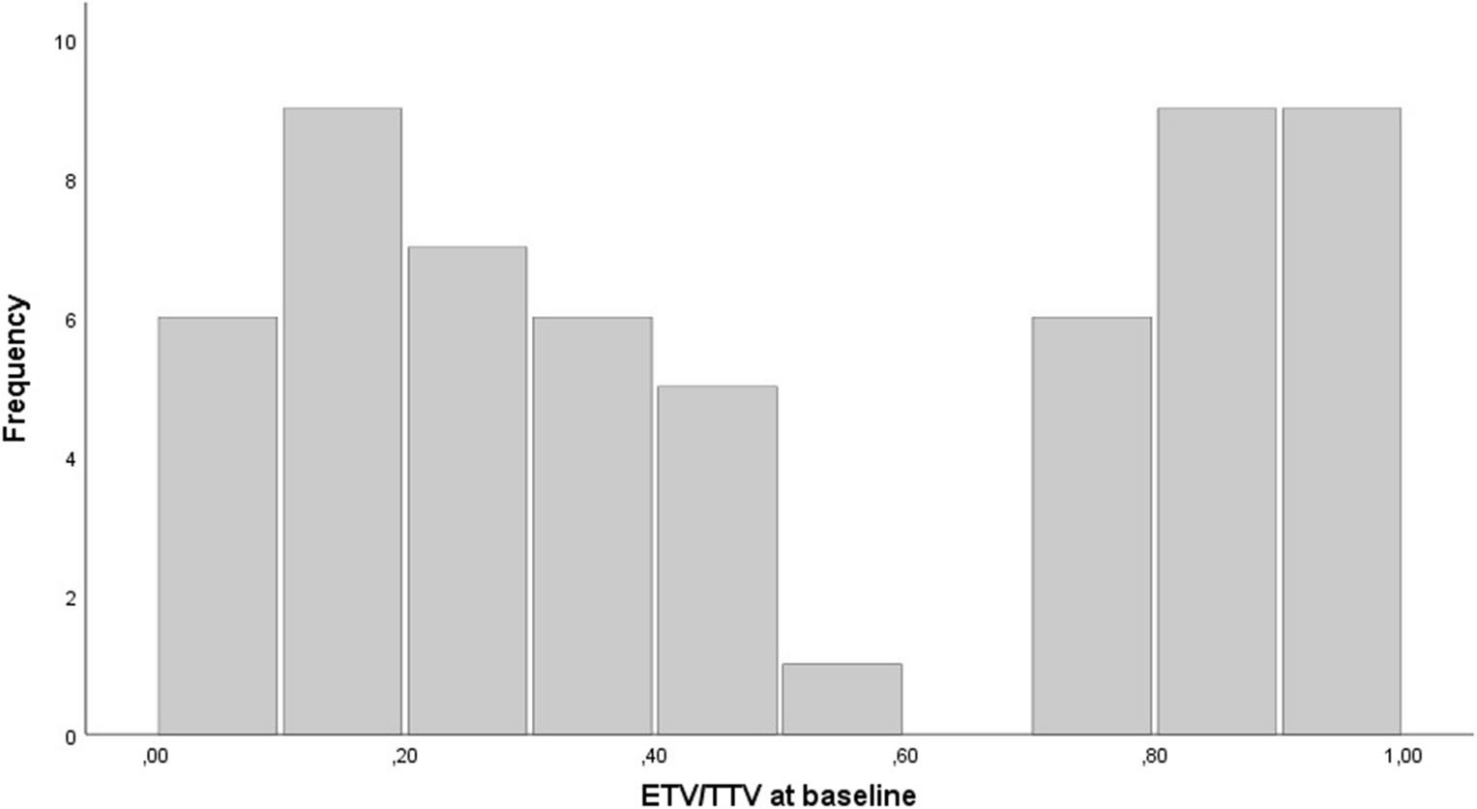
Frequency distribution of relative vascularization at baseline. The enhancing tumor volume/total tumor volume (ETV/TTV) ratio showed a pronounced bimodal distribution with frequency peaks between 10 and 30% and above 80% relative arterial tumor vascularization

**Fig. 4:**
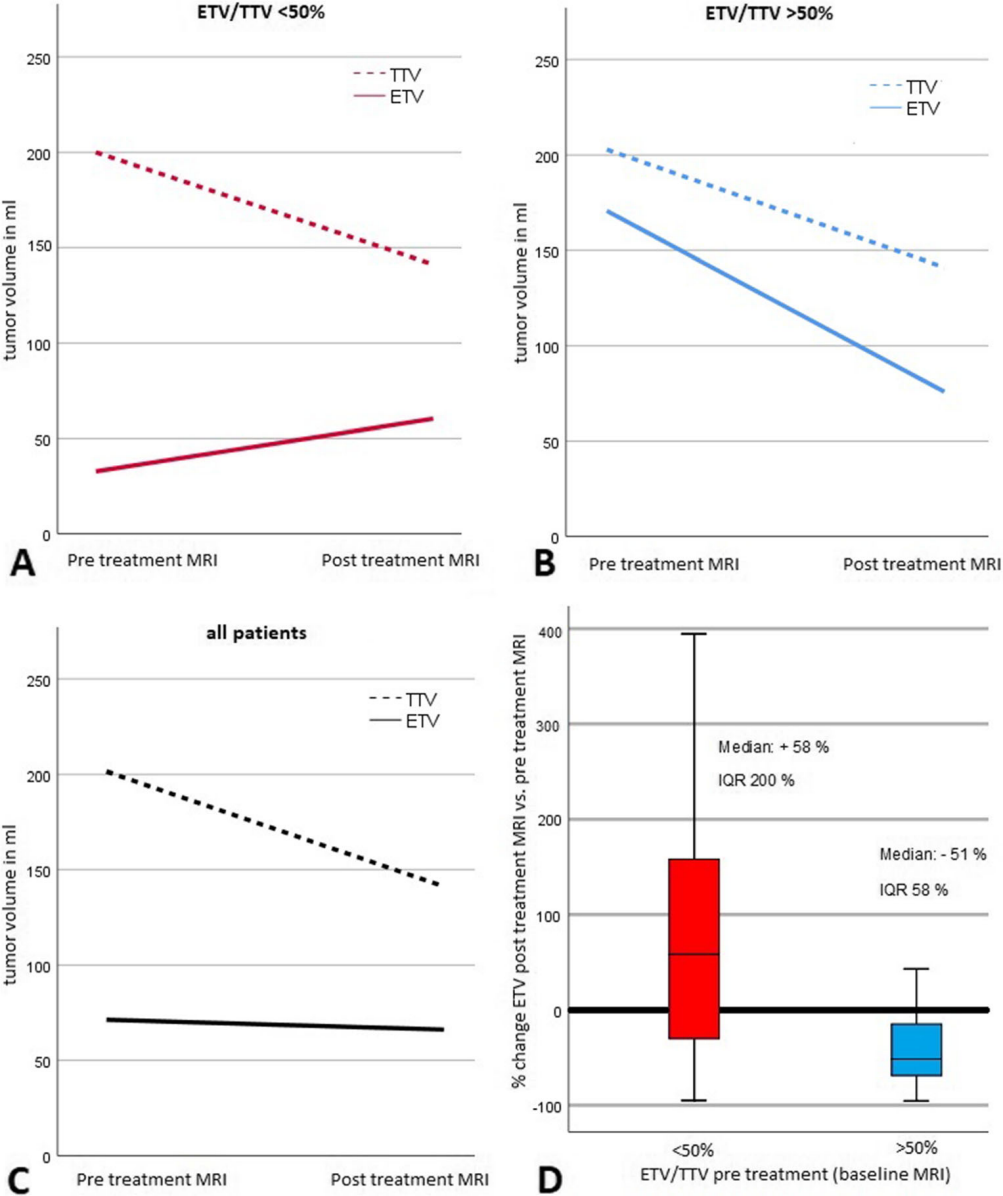
Impact of relative vascularization at baseline on later decrease in enhancing tumor volumes. Line charts in **A** to **C** depict the development of enhancing tumor volume (ETV) and total tumor volume (TTV) between baseline and follow-up MRI for patients with low tumor vascularization (ETV/TTV < 50% in baseline MRI), high tumor vascularization (ETV/TTV > 50% in baseline MRI), and the whole cohort. The change of median ETV over time in both subgroups is compared with boxplots in **D**

TTV and ETV on CE MRI both at baseline and 3 months after treatment were approximated by summarizing the volumes of a maximum of three dominant lesions in 58 patients (Table 2). The inter-reader agreeability of our unidirectional and volumetric MRI measurements as measured with the Pearson correlation coefficient (PCC) statistic varied between 0.93 and 0.99 (Supplement 3) which is in line with results from previous studies [22–24]. The measurements for target lesion TTV at baseline correlated significantly with total tumor volume assessment performed routinely before radioembolization with a PCC of 0.88 (*p* < 0.001). After transformation with the natural logarithm, baseline TTV and ETV followed a log-normal distribution. The non-logarithmic ETV/TTV ratio showed a pronounced bimodal distribution with frequency peaks between 10 and 30% and above 80% relative arterial tumor vascularization (Fig. 3). ROC analysis and Youden’s *J* statistic revealed an ETV/TTV threshold of 50% to be most suitable to stratify patients in two significantly different (*p* < 0.001) groups in terms of ETV change as assessed with the Mann–Whitney *U* test. Patients with a baseline ETV/TTV ratio greater than 50% (25/58 patients) showed a median reduction in ETV by 51% and less variability with an IQR of 58%. Patients with a ratio less than 50% (33/58 patients) had a median increase in ETV of 58% with an IQR of 200% (Fig. 4). Apart from a higher proportion of BCLC C patients in the group with a baseline ETV/TTV ratio < 50% (45% of the patients vs. 20% of the patients in the ETV/TTV ≥ 50% group), the two groups did not show significant differences with respect to baseline clinical variables (Supplement 4).

**Table 2:** Quantitative target lesion analysis on contrast-enhanced MRI before and after Y-90 radioembolization

Quantitative target lesion analysis (median, IQR)			
	Baseline	Follow-up	Significance
TTV in mL	201 (370)	141 (272)	*p* < 0.001
ETV in mL	71 (160)	66 (153)	*p* = 0.34
ETV/TTV	46 (68)	55 (59)	*p* = 0.34
ETV reduction in %	−15 (129)		

*ETV*, enhancing tumor volume; *IQR*, interquartile range; *ml*, milliliter; *TTV*, total tumor volume

### Prediction of survival based on qEASL response, baseline imaging, and clinical variables

**Fig. 5:**
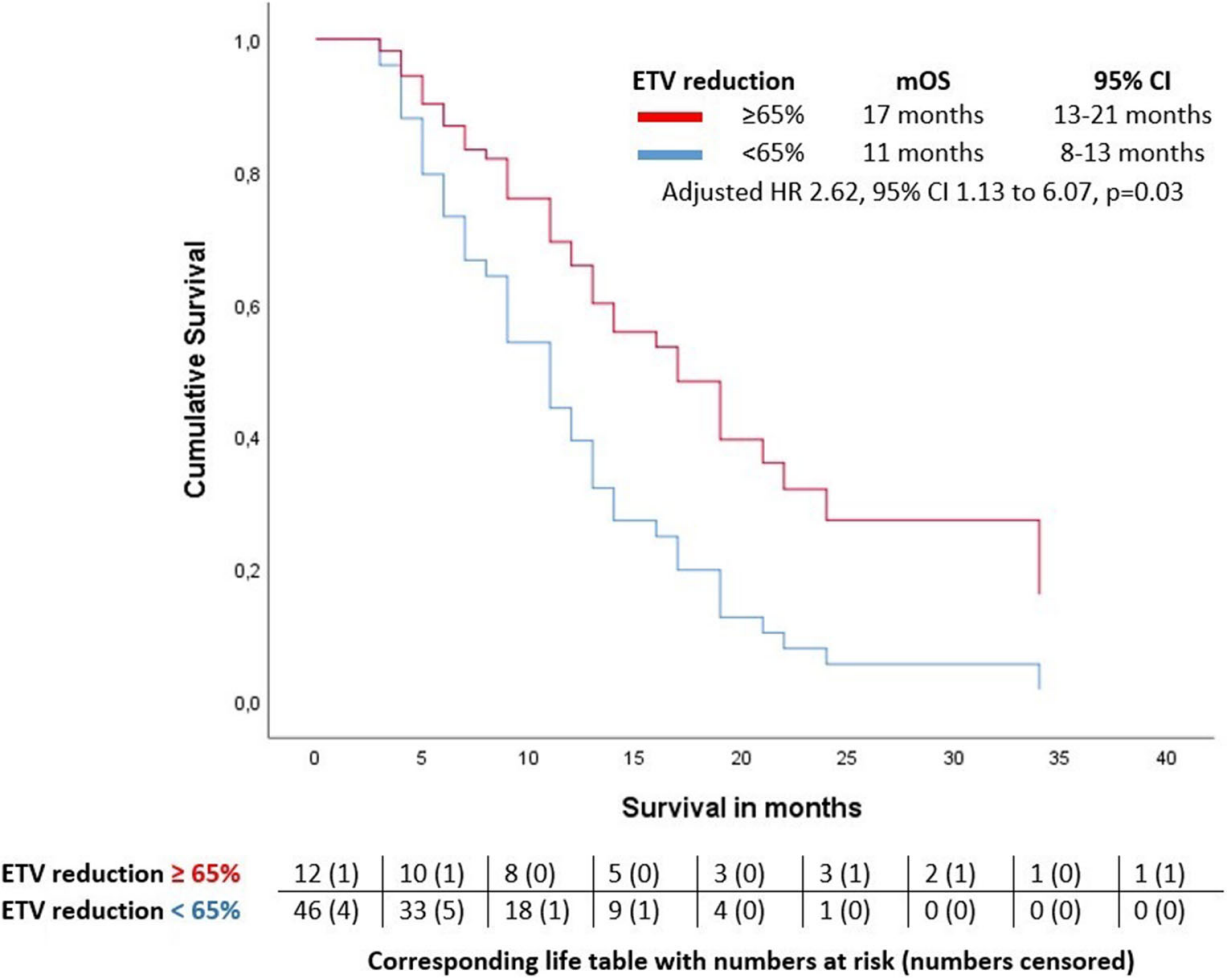
Comparison of survival times of MRI morphologic therapy responders and non-responders with Cox regression. Survival curves for the stratification into responders and non-responders who showed a reduction of enhancing tumor volume (ETV) greater or smaller than 65% in follow-up MRI. Median survival in non-responders was 11 months (95% confidence interval (CI) 8–14 months) and 17 months in responders (95% CI 13–21 months); the adjusted hazard ratio (HR) was 2.62. Numbers at risk and numbers censored are given at the beginning of the interval

The population included for tumor volumetry had a mOS of 11 months (95% CI 8–14 months). In univariable Cox regression, age above 70 years and the presence of liver cirrhosis significantly decreased patient survival; prior ablation, cTACE, or systemic therapy were no significant predictors, nor was response according to qEASL criteria on follow-up MRI with a *p* value of 0.06 (Table 3). In multivariable Cox regression, survival was best explained by a model that included qEASL response, the status on cirrhosis, and patient age above or below 70 years as predictors (chi-squared test, *p *= 0.001), whereas qEASL response was the single strongest predictor with a HR of 2.6 (*p *= 0.03) (Table 3). Survival curves for the stratification into MRI morphologic responders and non-responders are shown in Fig. 5. Median survival in non-responders was 11 months (95% CI 8–14 months) and 17 months in responders (95% CI 13–21 months).

**Table 3:** Univariable and multivariable Cox regression analyses

Variables	Univariable model		Multivariable model	
Demographics	HR	Sign.	HR	Sign.
Sex (male)	1.16	0.71		0.71
Age (years) % > 70	0.53	0.047	0.6	0.05
Liver disease				
BCLC tumor stage (B or C)	1.13	0.71		0.71
Hepatitis (yes/no)	0.67	0.21		0.21
Cirrhosis (yes/no)	0.39	0.01	0.4	0.01
Previous treatments				
TKI (yes/no)	1.34	0.40		0.40
Resection (yes/no)	1.34	0.44		0.44
TACE (yes/no)	0.64	0.2		0.20
Ablation (yes/no)	0.09	0.1		0.09
Radioembolization approach				
Sequential lobar therapy (yes/no)	1.63	0.13		0.13
Dose reduction (yes/no)	1.32	0.46		0.46
Applied dose/tumor volume (mBc/mL)	0.01	0.52		0.52
Tumor characterization				
Ln TTV (in mL)	0.98	0.9		0.90
Ln ETV (in mL)	0.99	0.94		0.94
ETV/TTV	0.96	0.94		0.94
ETV/TTV > 50%	0.93	0.81		0.81
Hepatopulmonary shunt in %	0.97	0.36		0.36
MRI response assessment				
Reduction in ETV by 65%	2.23	0.06	2.6	0.03
Performance of the multivariable model				
Significance			*p* = 0.001	
−2 log-likelihood (range, competing models)			249.5 (248-250)	

*BCLC*, Barcelona Clinic Liver Cancer stage; *ETV*, enhancing tumor volume; *HR*, hazard ratio; *Ln*, natural logarithm; *ml*, milliliter; *Sign.*, significance; *TACE*, transarterial chemoembolization; *TKI*, tyrosine kinase inhibitor; *TTV*, total tumor volume

### Prediction of qEASL response at baseline

Univariable and multivariable logistic regression analyses were performed to determine the impact of the abovementioned baseline variables on the likelihood of MRI morphologic qEASL response 3 months after Y-90 radioembolization. Variables with a *p* value smaller than 0.25 in univariable regression were further evaluated in multivariable regression analysis which did not apply to severity of liver disease, demographic data, or previous treatments (Table 4). In multivariable logistic regression analysis, Ln TTV, hepatopulmonary shunting, and an ETV/TTV ratio greater than 50% were significant predictors of MRI morphologic treatment response with *p *< 0.05. Elimination of individual predictors from the multivariable model did not improve model fit with respect to the information criteria and Nagelkerke’s pseudo-*R*^2^.

**Table 4:** Univariable and multivariable logistic regression analysis with respect to MRI morphologic tumor response at follow-up

Variables	Univariable model		Multivariable model	
Demographics	OR	Sign.	OR	Sign.
Sex (male)	0.70	0.59		
Age (years) > 70	0.91	0.85		
Liver disease				
BCLC tumor stage (C vs. B)	0.31	0.16		
Hepatitis (yes/no)	1.93	0.37		
Cirrhosis (yes/no)	1.03	0.96		
Previous treatments				
TKI (yes/no)	0.44	0.23		
Resection (yes/no)	0.56	0.41		
TACE (yes/ no)	2.19	0.35		
Ablation (yes/no)	4.30	0.18		
Radioembolization approach				
Sequential lobar therapy (yes/no)	2.31	0.25	5.8	0.07
Dose reduction (yes/no)	0.73	0.68		
Tumor characterization				
Ln TTV (in mL)	0.52	0.03	0.4	0.01
Ln ETV (in mL)	0.73	0.23		
ETV/TTV	4.39	0.15		
ETV/TTV > 50%	3.41	0.07	6.3	0.02
Hepatopulmonary shunt in %	1.08	0.23	1.3	0.01
Performance of the multivariable model				
Significance			*p* = 0.001	
Nagelkerke’s pseudo-*R*^2^ (range, competing models)			0.44 (0.30-0.44)	

*BCLC*, Barcelona Clinic Liver Cancer stage; *ETV*, enhancing tumor volume; *HR*, hazard ratio; *Ln*, natural logarithm; *ml*, milliliter; *Sign.*, significance; *TACE*, transarterial chemoembolization; *TKI*, tyrosine kinase inhibitor; *TTV*, total tumor volume

**Fig. 6:**
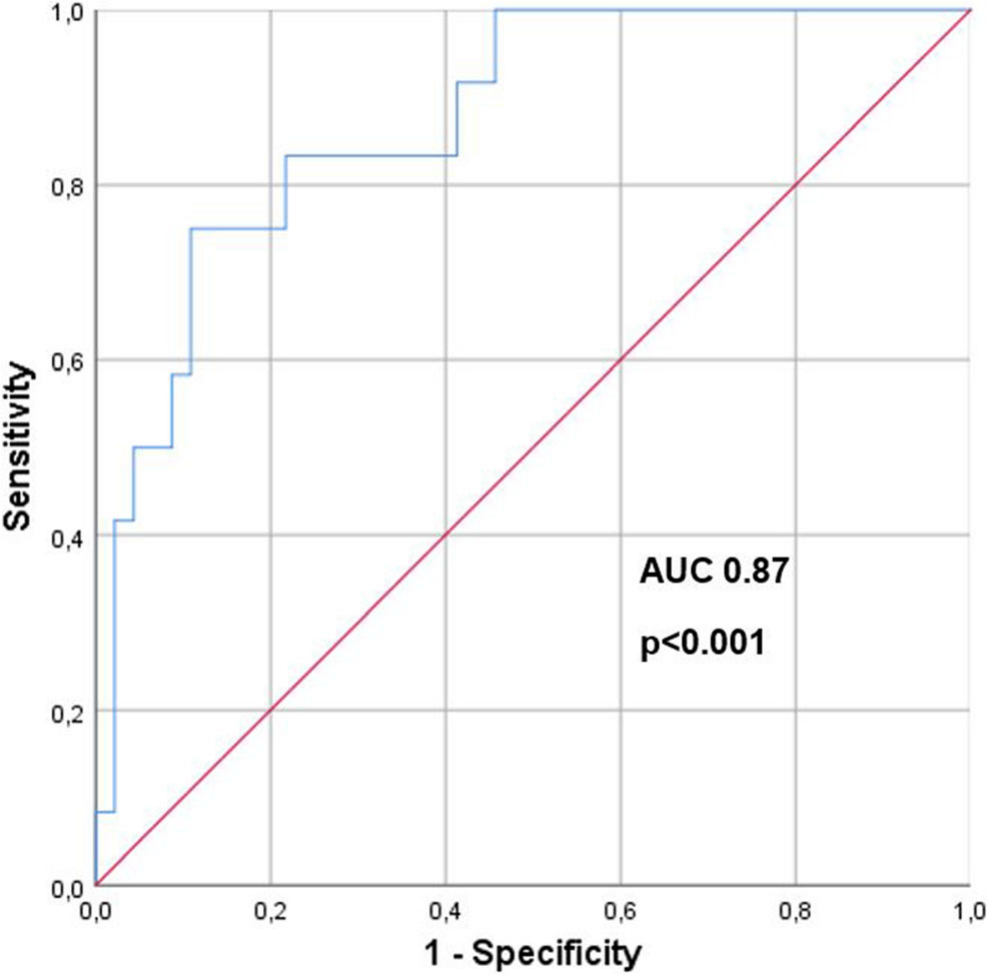
Analysis of the multivariable logistic regression model as binary classifier of MRI morphologic treatment response with ROC analysis and AUC. The performance of the multivariable logistic regression model as binary classifier was evaluated with receiver operating characteristic (ROC) analysis which revealed an area under the curve (AUC) of 87%. Significance for AUC differing from 0.50 was tested with the Mann–Whitney *U* test

The multivariable logistic regression model was statistically significant at *χ*^2^ [4] = 19.178 and *p *= 0.001. The model accounted for 44% (Nagelkerke’s pseudo-*R*^2^) of the variance in MRI morphologic treatment response which corresponded to a Cohen’s f of 0.89 and indicated a strong effect size (Table 4). The performance of the multivariable logistic regression model as binary classifier was verified with ROC analysis which revealed an area under the curve (AUC) of 87% (Fig. 6). An ETV/TTV ratio greater than 50% was the strongest predictor of qEASL response with an OR of 6.3.

## Discussion

This retrospective, single-center study demonstrates that a decrease of 65% ETV on follow-up imaging, response according to qEASL criteria, after Y-90 radioembolization enables the early prediction of significantly and relevantly improved patient survival. An ETV/TTV ratio greater 50% before treatment is the most important variable in a multivariable logistic regression model that predicts qEASL response at baseline at a high level of significance (*p* = 0.001) with an AUC of 87%. Administered activity in total and per tumor volume using BSA method did neither predict patient survival nor devascularization on follow-up imaging. The baseline ETV/TTV ratio of locally advanced, unresectable HCC follows a bimodal distribution with frequency peaks of either low (10–30%) or high (> 80%) relative arterial tumor vascularization.

The median survival of our study cohort was 11 months which is in accordance with data from the protocol population of the SARAH trial (mOS of 9.9 months) [13]. As opposed to the Y-90 radioembolization protocol of the SARAH trial which stipulated sequential liver treatment, 23 out of 58 patients in our study underwent a whole liver treatment approach in one session. Most of these patients were treated before 2012 when sequential therapy was not yet universally adopted in clinical practice. In our study cohort, the MRI morphologic, volumetric partial response threshold of 65% allowed for the separation of the survival curves into one of subsequently labeled non-responders with a median survival of 11 months and one of responders with a median survival of 17 months (*p *= 0.04). The so-called qEASL criteria were already proven prognostically useful in HCC collectives that underwent cTACE. Data by Lewandowski et al which demonstrated partial or complete response to be the common variable in patients with an OS longer than 3 years after Y-90 radioembolization further corroborates the rationale for this threshold [21, 25, 26]. Interestingly, these results resemble the difference in mOS of 6.1 and 14.1 months that Hermann et al from the SARAH trial group demonstrated after stratification according to absorbed tumor dose lesser or greater than 100 Gy in Tc-99m-macroaggregated albumin SPECT/CT-based dosimetry [27]. The correlation of baseline arterial tumor vascularization, tumor–to–normal liver ratio on Y-90 SPECT, and tumor devascularization on follow-up imaging was demonstrated in a recent publication; an in-depth analysis of this matter was outside the scope of this study [16].

Given the significant impact of tumor devascularization on patient survival, we believed this to be a more robust surrogate measure for the efficacy and benefit of Y-90 radioembolization than mOS itself in a patient collective as complex and frail as candidates for radioembolization. Therefore, we investigated baseline imaging and clinical variables as potential predictors of devascularization. The bimodal distribution of baseline ETV/TTV ratios observed in locally advanced, unresectable HCC is probably reflective of tumor biology. The tendency to either preserve high vascularity without development of central necrosis or rapidly turn relatively hypovascular is consistent with observations made in HCC TACE collectives that were assessed with the same quantitative imaging approach [25]. Ln TTV and Ln ETV showed an inverse correlation with treatment response, probably because larger tumors have a greater tendency to turn hypoxic and develop necrosis. The much more heterogeneous effect of Y-90 radioembolization on ETV post-treatment below a threshold of 50% ETV/TTV indicates that probably a minimum relative arterial vascularization or rather tumor-to-liver ratio of dose distribution is a prerequisite for Y-90 radioembolization to consistently cause contiguous tissue necrosis. This might at least be true if the radiation dose is calculated in a traditional fashion based on BSA, liver volume, and tumor volume. A more sophisticated approach of using pre-therapeutic Tc-99m-MAA SPECT to estimate later intratumoral Y-90 distribution for individualized dose escalation showed promising preliminary results and might help to compensate for rather scarce tumor vascularity [1, 17, 18]. Hepatopulmonary shunting is known to correlate with tumor vascularization which might explain its positive correlation with treatment response [28, 29]. Nevertheless, this finding is surprising as other studies demonstrated increased hepatopulmonary shunting to be associated with poorer outcomes [30–32]. A possible explanation may be that in our collective, a positive correlation of shunting and later tumor-to-liver ratio of dose distribution outweighs the impact of dose reduction above a threshold of 10% on intratumoral dose density and subsequent devascularization on follow-up imaging. Lobar treatment also correlated positively with treatment response, probably due to a better toxicity profile [33].

An exemplary multivariable logistic regression model that incorporated baseline MRI tumor segmentation data and controlled for clinical variables had a remarkably strong effect size with a Cohen *f* of 0.89 and predicted MRI morphologic treatment response very well. Such a prediction model is how we expect baseline imaging and clinical information to be extremely helpful in improving patient selection and treatment outcomes. Although we systematically controlled for confounding and overfitting, the model at hand cannot be used for generalization. It merely helps to illustrate the impact baseline imaging can have in a potential prediction model of response. Before we could run any model, the crucial step of cross-validation aside, one would first have to decide whether potential responders or non-responders of radioembolization were to be predicted which, not least, is an ethical matter. Second, one would have to agree on an acceptable sensitivity and specificity of threshold values for the classification as either responders or non-responders. All these steps of model specification we purposely left out because they are futile as long as a given model cannot be cross-validated. In clinical routine and with a properly cross-validated model, the values of predictor variables for an individual patient could be entered into the equation of the model. With respect to pre-defined thresholds, certain scores would inform the decision to treat patients with radioembolization, to consider them for individualized dose escalation protocols, or to evaluate them for an entirely different treatment strategy.

There are a number of considerable limitations to the generalizability of our results. First, the patient cohort was too small to perform model building and cross-validation at the same time. Although we limited the number of predictors to a maximum of four with a study population of 58 patients, we expect substantial overfitting of our model. Second, the original patient population was very inhomogeneous especially in terms of whole liver versus sequential therapy, preinterventional imaging modalities, and timing of follow-up imaging. This accounts for the relatively small proportion of patients that could be included for further evaluation. Third, there were no SPECT Y90 tumor absorbed-dose metrics performed in our cohorts which would have been very insightful to correlate with relative vascularization at baseline and could have further strengthened our hypotheses.

In summary, we present a novel approach to identify promising candidates for Y-90 radioembolization by using tumor volumetry, clinical variables, and regression modeling techniques for the prediction of tumor devascularization and associated survival benefits. This promising concept must be cross-validated in future studies in order to conceive generalizable prediction models for clinical routine. The predictive potential of tumor vascularity for later tumor-to-liver ratio of Y-90 deposition merits further investigation and should be evaluated for other potential indications of Y-90 radioembolization.

## Supplementary Information

ESM 1 (DOCX 47 kb)

## Abbreviations

BSA: Body surface area
CE: Contrast enhanced
ETV: Enhancing tumor volume
HCC: Hepatocellular carcinoma
HR: Hazard ratio
IQR: Interquartile range
Ln: Natural logarithm
mOS: Median overall survival
OR: Odds ratio
OS: Overall survival
PCC: Pearson correlation coefficient
qEASL: Quantitative European Association for the Study of the Liver (criteria)
ROC: Receiver operating characteristic
TACE: Transcatheter arterial chemoembolization
TTV: Total tumor volume
Y90: Yttrium-90(-radioembolization)

## Declarations

### Guarantor

The scientific guarantor of this publication is PD Dr. Gero Wieners.

### Statistics and biometry

Juliane Hardt kindly provided statistical advice for this manuscript.

### Methodology

• retrospective

• observational

• performed at one institution
