# Correction: Yttrium-90 radioembolization for unresectable hepatocellular carcinoma: predictive modeling strategies to anticipate tumor response and improve patient selection

**DOI:** 10.1007/s00330-025-11907-4

**Published:** 2025-09-11

**Authors:** Willie Magnus Lüdemann, Johannes Kahn, Daniel Pustelnik, Juliane Hardt, Georg Böning, Martin Jonczyk, Holger Amthauer, Bernhard Gebauer, Bernd Hamm, Gero Wieners

**Affiliations:** 1 Department of Radiology, Campus Virchow Klinikum, Charité, Augustenburger Platz 1, 13353 Berlin, Germany; 2 Department of Biometry, Epidemiology and Information Processing, WHO Collaborating Centre for Research and Training for Health in the Human-Animal-Environment Interface, University of Veterinary Medicine Hannover (Foundation), Buenteweg 2, 30559 Hanover, Germany; 3 Department of Nuclear Medicine, Augustenburger Platz 1, 13353 Berlin, Germany

**Correction to: European Radiology**

10.1007/s00330-022-08585-x published online 01 March 2022.

In this article, the middle name of author Willie Magnus Lüdemann was left out by mistake and has been added since.

The original article has been corrected.

