# Supplementary material for: Yttrium-90 radioembolization for unresectable hepatocellular carcinoma: predictive modeling strategies to anticipate tumor response and improve patient selection
Source: Eur Radiol. 2022 Mar 1;32(7):4687–98. doi: 10.1007/s00330-022-08585-x (PMC9213379; doi:10.1007/s00330-022-08585-x)
Supplement: Supplementary file 1 — (DOCX 47 kb) [file 330_2022_8585_MOESM1_ESM.docx]

**Supplements**

1: Institutional protocol for Y-90 radioembolization

One to two weeks before treatment, all patients were assessed angiographically for vascular anatomy and with Tc-99m-macroaggregated albumin (MAA) SPECT/CT to estimate shunting to the lung and exclude microsphere deposition to the gastrointestinal tract. After local anesthesia with lidocaine 1%, a 5F vascular sheath was placed preferably in the right common femoral artery in Seldinger-technique via a 0.035-inch guide wire. An overview angiography of the celiac trunc and the superior mesentery artery was generated with a 5-F Cobra (Radifocus, Terumo Europe NV) or a 5-F SOS Omni Selective catheter (Soft-Vu, Angiodynamics). Afterwards, a microcatheter (Cantata 2.5 F or MicroFerret-18 3 F, Cook Medical) was advanced into the proper hepatic artery. Additional DSA runs allowed for analysis of hepatic artery anatomy, HCC tumour blushes and aberrant arterial blood supply. If necessary, coil-embolization of the gastroduodenal artery, the right gastric artery or any accessory artery was carried out to avoid later off-target embolization. After selective placement of a microcatheter in both the right and the left liver lobe, scintigraphy was performed with 120–200 MBq of technetium-99m-labelledmacroaggregated albumin (99mTc-MAA) to assess hepatopulmonary shunting. Y-90 radioembolization was considered feasible if there was no relevant abdominal off-target distribution of 99mTv-MAA and if later pulmonary radiation exposure due to hepatopulmonary shunting did not exceed 30 Gy. The radiation dose of Y90 was calculated based on the body surface area and the tumour burden of each lobe according to the equation below. If the hepatopulmonary shunt fraction of 99mTc-MAA was between 10 and 15% the total dose for Y-90 radioembolization was reduced by 20%, for a shunt fraction between 15 and 20% the dose could be reduced by up to 40%.

$$Radiation Dose =Body surface area in m^{2} - 0,2 + \frac{\frac{Tumour Volume}{Treated Liver Volume} in \%}{100}$$

Therapy was performed in a separate session according to the angiographic standard protocol above by placement of a microcatheter in a lobar liver artery and application of the calculated dose. Depending on liver function, ECOG overall performance status, tumour burden and hepatopulmonary shunting the whole liver was treated in a single session or the right and left liver lobes were treated in a sequential fashion in accordance with the tumour board decision.

**2: Characteristics of patients included and excluded for segmentation**

| Demographics | | Included | | Excluded | Sign. |
| --- | --- | --- | --- | --- | --- |
| Number of patients, n (%) | | 58 (37%) |  | 100 (63%) |  |
| Mean age (years), mean (SD) | | 66 (8.7) |  | 66.4(11.5) | 0.44 |
| Sex: male / female (% male) | | 46/12 (79%) |  | 76/24 (76%) | 0.63 |
| Median survival in months (95% CI)* | | 11 (8-14) |  | 8 (7-11) | 0.22 |
| Liver disease, n (%) | | | | | |
| BCLC stage | B | 38 (65%) | | 70 (70%) | 0.56 |
|  | C | 20 (35%) | | 30 (30%) |  |
| Child Pugh Score | A | 56 (96%) | | 87 (87%) | 0.05 |
|  | B | 2 (4%) | | 13 (13%) |  |
| Cirrhosis | | 39 (67%) |  | 62 (62%) | 0.67 |
| Hepatitis B or C | | 21 (36%) |  | 35 (35%) | 0.88 |
| Portal vein thrombosis | | 15 (26%) | | 30 (30%) | 0.58 |
| Previous treatments, n (%) (possible multiple therapies) | | | | | |
| Resection | | 10 (17%) |  | 24 (23%) | 0.32 |
| TACE | | 16 (28%) |  | 17 (17%) | 0.12 |
| Brachytherapy | | 15 (26%) |  | 16 (16%) | 0.13 |
| RFA | | 4 (5%) |  | 7 (7%) | 0.98 |
| PEI | | 1 (2%) |  | 0 |  |
| Sorafenib | | 16 (28%) |  | 31 (31%) | 0.57 |
| Other systemic therapy | | 2 (3%) |  | 7 (7%) | 0.35 |
| Specifics of Y-90 radioembolization | | | | | |
| Mean hepatopulmonary shunt in % (SD) | | 8.6% (4.7%) |  | 10.61% (5.96%) | 0.025 |
| Median tumour volumes in ml (IQR) | | 209 (326) |  | 287 (603) | 0.223 |
| Median activity in mBq (IQR) | | 1.7 (0.53) |  | 1.48 (0.62) | 0.006 |
| *Kaplan-Meier estimator  Abbreviations:  BCLC: Barcelona Clinic liver cancer stage (BCLC)  CI: confidence interval  ETV: enhancing tumour volume  IQR: interquartile range  mBq: megabecquerel  ml: milliliter  PEI: percutaneous ethanol injection  RFA: radiofrequency ablation  SD: standard deviation  Sign.: significance  TACE: transarterial chemoembolization  TTV: total tumour volume | | | |  |  |


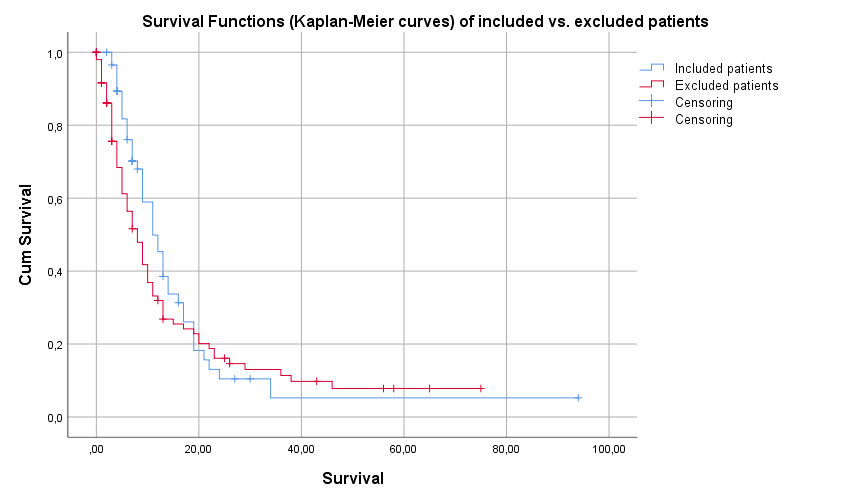


**3: Reproducibility data MRI measurements: agreement of 2 independent measurements (Pearson Correlation Coefficient PCC)**

**MRI measurements at baseline**

| **Measurements** | **PCC** |
| --- | --- |
| Longest diameter | **0.997** |
| Shortest diameter | **0.986** |
| Total tumour volume (TTV) | **0.989** |
| Enhancing tumour volume (ETV) | **0.953** |
| ETV/TTV | **0.927** |

**MRI measurements after treatment (follow-up)**

| **Measurements** | **PCC** |
| --- | --- |
| Longest diameter | **0.978** |
| Shortest diameter | **0.960** |
| Total tumour volume (TTV) | **0.984** |
| Enhancing tumour volume (ETV) | **0.977** |
| ETV/TTV | **0.957** |

**4: Extended patient characteristics according to ETV/TTV <> 50%**

| Demographics | | | Total | | ETV/TTV < 50% | ETV/TTV ≥ 50% | Sign. |
| --- | --- | --- | --- | --- | --- | --- | --- |
| Number of patients (%) | | | 58 |  | 33 (57%) | 25 (43%) |  |
| Mean age (years), mean (SD) | | | 66 (8.7) | | 65 (9.3) | 66 (8.5) | 0.70 |
| Sex: male / female, n (% male) | | | 46/12 (79%) |  | 27/6 (82%) | 19/6 (76%) | 0.59 |
| Median survival in months (95% CI)* | | | 11 (8-14) |  | 11 (8-14) | 13 (11-15) | 0.81 |
| Liver disease, n (%) | | | | | | | |
| BCLC stage | | B | 38 (66%) | | 18 (55%) | 20 (80%) | 0.04 |
|  |  | C | 20 (34%) | | 15 (45%) | 5 (20%) |  |
| Child Pugh Score | | A | 56 (97%) | | 33 (100%) | 23 (92%) | 0.10 |
|  |  | B | 2 (3%) | | 0 | 2 (8%) |  |
| Cirrhosis | | | 39 (67%) |  | 23 (70%) | 16 (64%) | 0.65 |
| Hepatitis | | | 21 (36%) |  | 15 (45%) | 6 (24%) | 0.09 |
| Portal vein thrombosis | | | 15 (26%) | | 11 (33%) | 4 (16%) | 0.14 |
| Previous treatments, n (%) (possible multiple therapies) | | | | | | | |
| Resection | | | 10 (17%) |  | 7 (21%) | 3 (12%) | 0.36 |
| TACE | | | 16 (28%) |  | 9 (27%) | 7 (28%) | 0.95 |
| Ablation (brachytherapy, RFA, PEI) | | | 20 (34%) |  | 12 (36%) | 8 (32%) | 0.73 |
| Sorafenib therapy | | | 16 (28%) |  | 11 (33%) | 5 (20%) | 0.26 |
| Specifics of Y-90 radioembolization | | | | | | | |
| Mean hepatopulmonary shunt in % (SD) | | | 8.6 (4.7) | | 9.6 (5.5) | 7.6 (3.6) | 0.41 |
| Median tumour volumes in ml (IQR) | | | 209 (326) | | 207 (317) | 224 (421) | 0.62 |
| Median activity in mBq (IQR) | | | 1.7 (0.53) | | 1.6 (0.54) | 1.8 (0.54) | 0.65 |
| Tumour segmentation, median (IQR) | | | | | | | |
| TTV in ml | baseline | | 201 (370) | | 174 (374) | 202 (273) | 0.81 |
|  | follow-up | | 141 (272) | | 141 (266) | 141 (289) | 0.74 |
| ETV in ml | baseline | | 71 (160) | | 32 (67) | 169 (255) | 0.001 |
|  | follow-up | | 66 (153) | | 60 (109) | 94 (260) | 0.59 |
| ETV/TTV ratio in % | baseline | | 46 (68) | | 22 (25) | 87 (21) | 0.001 |
|  | follow-up | | 55 (59) | | 47 (62) | 68 (38) | 0.05 |
| ETV change in % |  | | -15 (129) | | 60 (206) | -51 (59) | <0.001 |
| *Kaplan-Meier estimator  Abbreviations:  BCLC: Barcelona Clinic liver cancer stage (BCLC)  CI: confidence interval  ECOG: Eastern Cooperative Oncology Group performance (ECOG)  ETV: enhancing tumour volume  IQR: interquartile range  mBq: megabecquerel  ml: milliliter  PEI: percutaneous ethanol Injection  RFA: radiofrequency ablation  SD: standard deviation  TACE: transarterial chemoembolization  TTV: total tumour volume | | | | |  |  |  |
